# Theranostics Implementation: Opportunities and Challenges

**DOI:** 10.1055/s-0045-1812103

**Published:** 2025-10-07

**Authors:** Kunthi Pathmaraj, Andrew M. Scott

**Affiliations:** 1 Department of Molecular Imaging and Therapy, Austin Health, Melbourne, Australia; 2 Olivia Newton-John Cancer Research Institute, Melbourne, Australia; 3 School of Cancer Medicine, La Trobe University, Melbourne, Australia; 4 School of Health and Biomedicine, RMIT University, Melbourne, Australia; 5 Faculty of Medicine, University of Melbourne, Melbourne, Australia

Theranostics is emerging as one of the most promising new approaches to cancer therapy, with recently approved indications and over 500 active clinical trials currently underway in patients with solid tumors and hematologic malignancies. This extends from early phase academic trials to industry-sponsored, multicenter, multinational trials with β and α particle emitting radioisotopes linked to targeting moieties. 1 The development of combined imaging and treatment approaches dates back to the development of ^131^ I therapy by Saul Hertz, and this treatment has been adopted globally for treatment of hyperthyroidism and thyroid cancer. 1 2 More recent developments in theranostic treatment have been for selective delivery of radiolabeled microspheres for liver cancer, ^131^ I-MIBG for neuroblastoma, paraganglioma, and pheochromocytoma tumors, ^177^ Lu-DOTATAE for neuroendocrine tumors, and ^177^ Lu-PSMA for metastatic prostate cancer. 1 2 3 4 5 6 While the development and approval of theranostics has advanced dramatically over the past 10 years, the availability of these treatments has been limited to mainly high-income countries and has been the subject of a recent Lancet Oncology Commission which outlined the challenges and opportunities for theranostics at a global level. 2

In this special edition on theranostics, the key issues that are impacting on access and availability of theranostics worldwide are reviewed, as well as the required expertise and resources to implement and maintain successful theranostic programs. Brink et al provide a perspective on the challenges and approaches to theranostics implementation at a global level, and highlight the disparity in access and availability in many countries. 6 They also provide information on the important role of the International Atomic Energy Agency (IAEA) in addressing the key areas impacting on theranostics availability, including workforce, guidelines, regulatory approaches, and radiopharmaceutical access. The challenges in providing theranostics services is particularly relevant in low- and middle-income countries (LMICs), and Lawal et al provide an important perspective on how LMICs can implement theranostic programs, thus ensuring broader availability for patients. 7 The lack of a suitably trained workforce is an issue in high-income countries and LMICs, and guidelines for training nuclear medicine staff, as well as requirements for theranostics centers, have been addressed in recent high-level reviews. 8 9 10

The development of radiopharmaceuticals suitable for both imaging and therapy, and access to these novel radiopharmaceuticals, is a crucial part of the ability to implement a theranostics service. 2 11 12 While therapeutic β-particle emitting radioisotopes play a key role in most currently approved theranostics, α-particle emitting radioisotopes are also emerging as highly successful in treating advanced cancers. 1 2 11 In this edition, Bolin and Groves provide a comprehensive overview of α-particle therapies approved and in development, and the practical aspects of handling both α-particle radiopharmaceuticals and patients after treatment. 13 Radiation safety is an essential component of the practice of theranostics, and guidelines for the safe and effective treatment of patients and management of staff and the public remain a key focus for this treatment approach. 2 6 12 Procedure guidelines are also required to ensure patient selection, and treatment for approved indications are performed to an appropriate standard. 2 6 14 15

Dosimetry is also a key requirement for effective delivery of radiopharmaceuticals for therapy. 2 6 Bailey et al provide a detailed overview of dosimetric approaches to radioembolization therapy including practical implementation and patient workflow. 16 The approach to dosimetry in pediatric patients is addressed in two additional papers in this edition. London et al provide a comprehensive overview of dosimetry approaches to the delivery of theranostic treatment in pediatric patients with neuroblastoma, with cases to demonstrate the implementation of this approach. 17 Trpezanovski et al outlines the implementation of dosimetry to guide treatment of a young patient with a bronchial tumor, to achieve improved outcomes for the patient. 18 The use of personalized dosimetry for patient selection and treatment will be an increasingly important component of theranostics practice in the future. 2

The establishment of the safety and efficacy of theranostic treatments is essential to obtain both regulatory and funding approvals, and both academic- and industry-sponsored clinical trials are a key component of the requirement to generate the evidence required for such approvals. 3 4 5 Pathmaraj and Lee provide an overview of the approaches to conducting clinical trials in theranostics, including clinical trial networks and the need for multidisciplinary care teams. 19 A highly successful example of an academic clinical trial network is the Australasian Radiopharmaceutical Trials Network (ARTnet), and Francis et al provide a detailed perspective on the establishment of ARTnet and the successful multicenter theranostics trials conducted, which have led to regulatory approvals. 20 This type of academic, multicenter clinical trial network is being established in many countries and regions to facilitate evidence of theranostics safety and efficacy in different populations and is complementary to the large multicenter industry-sponsored studies which are integral to the development of many new theranostic treatments.

The implementation of theranostics at a country level is often challenging, and different parts of a country may find difficulties in staffing and logistics for access of radiopharmaceuticals and delivery of treatments. In this edition, Steyn et al outline the development of a national theranostics program for peptide-receptor radionuclide therapy, which has been highly successful and may be a template for similar programs in many other countries. 21 Morigi and McGavin provide an overview of a successful implementation of a positron emission tomography imaging service in a remote country location, highlighting the strategies required to achieve this outcome. 22 The clinical implementation of theranostics is one of the greatest challenges for nuclear medicine at a global level, but brings the potential for transformative impact for our field. The opportunities for theranostics and approaches for implementation outlined in this edition will contribute to highly impactful outcomes for our patients and families.
